# Altered Social Behaviours in Neurexin 1α Knockout Mice Resemble Core Symptoms in Neurodevelopmental Disorders

**DOI:** 10.1371/journal.pone.0067114

**Published:** 2013-06-28

**Authors:** Hannah Mary Grayton, Markus Missler, David Andrew Collier, Cathy Fernandes

**Affiliations:** 1 Social, Genetic and Developmental Psychiatry Centre, Institute of Psychiatry, King's College London, De Crespigny Park, London, United Kingdom; 2 Institute of Anatomy and Molecular Neurobiology, Westfälische Wilhelms-University, Münster, Germany; 3 Discovery Neuroscience Research, Eli Lilly and Company Ltd, Erl Wood, Windlesham, Surrey, United Kingdom; Roma Tre University, Italy

## Abstract

**Background:**

Copy number variants have emerged as an important genomic cause of common, complex neurodevelopmental disorders. These usually change copy number of multiple genes, but deletions at 2p16.3, which have been associated with autism, schizophrenia and mental retardation, affect only the neurexin 1 gene, usually the alpha isoform. Previous analyses of neurexin 1α (Nrxn1α) knockout (KO) mouse as a model of these disorders have revealed impairments in synaptic transmission but failed to reveal defects in social behaviour, one of the core symptoms of autism.

**Methods:**

We performed a detailed investigation of the behavioural effects of Nrxn1α deletion in mice bred onto a pure genetic background (C57BL/6J**)** to gain a better understanding of its role in neurodevelopmental disorders. Wildtype, heterozygote and homozygote Nrxn1α KO male and female mice were tested in a battery of behavioural tests (n = 9–16 per genotype, per sex).

**Results:**

In homozygous Nrxn1α KO mice, we observed altered social approach, reduced social investigation, and reduced locomotor activity in novel environments. In addition, male Nrxn1α KO mice demonstrated an increase in aggressive behaviours.

**Conclusions:**

These are the first experimental data that associate a deletion of Nrxn1α with alterations of social behaviour in mice. Since this represents one of the core symptom domains affected in autism spectrum disorders and schizophrenia in humans, our findings suggest that deletions within NRXN1 found in patients may be responsible for the impairments seen in social behaviours, and that the Nrxn1α KO mice are a useful model of human neurodevelopmental disorder.

## Introduction

Neurexins are a family of mostly presynaptic proteins [1] that form trans-synaptic complexes with postsynaptic neuroligins and other binding partners [2]–[3], allowing them to shape important synaptic functions [4]. Mammals contain three neurexin genes (NRXN1-3), each encoding two major isoforms, extracellularly longer α-neurexins and shorter β-neurexins. Through the presence of alternative promoters and alternative splicing at five conserved sites, potentially hundreds of different NRXN transcripts could be produced [3]. It has been reported that splicing patterns depend on neuronal activity [5]–[6], a process that may be highly regulated [7].

Mice with single and multiple deletions of the α-isoforms of all three NRXN genes demonstrated an essential role in synaptic transmission because spontaneous and evoked vesicle release are decreased in knockout (KO) animals [4], [8], indicating that neurexins may work to couple extracellular synaptic interactions to the intracellular organization of the presynaptic secretory apparatus. Deletion of α-neurexins was shown to impair neurotransmitter release which is dependent on N- and P/Q-type Ca^2+^ channels [4], [9], suggesting that α-neurexins are involved in regulating the function of these ion channels [9]–[10].

Numerous studies have reported associations between hemizygous exonic deletions within the NRXN1 gene (2p16.3) and neurodevelopmental disorders, including intellectual disability, developmental delay, autism and schizophrenia [11]–[17]. Furthermore, associations between homozygous deletions and Pitt-Hopkins-like Syndrome [18] and early-onset, severe, complex epilepsy [19] have been observed. Most deletions affect the 5′ exons of the alpha isoform of NRXN1 but some extend into exons encoding β-neurexin [11], [16], although it is not clear if these have a similar or more severe phenotype [20]. In Nrxn1α KO mice, two studies have observed behavioural alterations: Decreases in prepulse inhibition (PPI) of the startle response, an impairment in nest building activities, and an improvement in motor learning [21], and sex-dependent increases in response to novelty and accelerated habituation to novel environments [22] were reported. However, these studies tested mice maintained on a mixed genetic background (C57BL6/SV129), raising the possibility that differences in complex social behaviour, expected from deletions in this gene [11]–[17], were masked due to the contribution of genetic background effects [23]–[26]. Here, we thus assessed behaviours in Nrxn1α KO mice that had been backcrossed on to a single genetic background (C57BL/6J), and analysed phenotypes of relevance to human disorders [27].

## Materials and Methods

### Mice

Adult male and female Nrxn1α KO mice were generated as described [4]. Since these mice were previously maintained on a C57BL6/SV129 mixed genetic background, we subjected the line to 8 generations of backcrossing to C57BL/6J mice to transfer the knockout allele onto a standard C57BL/6J genetic background. From the offspring of the F8 pairing, Nrxn1α heterozygote mice were crossed together to generate the test mice (wildtype (+/+), Nrxn1 heterozygote KO (+/−) and homozygote KO (−/−) mice; n  = 9–16 per genotype, per sex). All mice were individually housed one week prior to testing with *ad libitum* access to water and food (see Materials S1 for detailed information). All housing and experimental procedures were performed in compliance with the local ethical review panel of King’s College London, and the U.K. Home Office Animals Scientific Procedures Act 1986. The work was carried out under licence (PPL: 70/7184) and all efforts were made to minimize animal suffering and to reduce the number of animals used.

### Behavioural Testing

Mice were ten weeks old at the start of testing and tests were recorded using a camera positioned above the test arenas and movement of each mouse tracked using EthoVision software (Noldus Information Technologies bv, Wageningen, The Netherlands; http://www.noldus.com/site/doc200403002). Further more detailed information about the protocols for all behavioural tests performed can be found in Materials S1.

#### Spontaneous locomotor activity

The homecage task was performed as described [28] except that locomotor activity was recorded at three 1 hour periods (namely 12 pm, 1 am, and 11 am the following morning).

#### Anxiety tasks

The open field, light/dark box and elevated plus maze were performed essentially as described [28].

#### Cognitive tasks

A range of cognitive tasks were carried out on the mice, including novel object discrimination, Morris water maze and delayed matching-to-place (DMP). Novel object discrimination task, performed as described [29], except that both short and long term memory were investigation using inter-trial intervals of 1 and 24 hours respectively. Morris water maze was performed essential as described [28], however mice were run in squads of 6 mice/squad, each mouse underwent four trials per day, mice were tested for 10 consecutive days and a probe task was run on the last day to assess the retention of spatial memory. DMP was carried out in the Morris water maze, as described [30], except mice underwent 8 trials/day for 7 days, the platform location was changed each day in a random manner, and the maximum trial length was 90 s. The reduction in latencies to find the platform between the first and subsequent trials is referred to as ‘saving time’ and is used as an index of working/episodic-like memory. (Additional pre-training was not carried out as mice had received extensive training during the Morris water maze testing prior to the DMP).

#### Social tasks

The three-chamber social approach task and the social investigation task were both performed. These were carried out essentially as described [31]–[32]. However for the three-chamber social approach task the equipment was not automated, instead the Ethovision tracking system was used to monitor the movement of the mice throughout the three chambers. For the social investigation task, test mice underwent two tests with adult and juvenile conspecifics, respectively. If prolonged periods of aggression was seen throughout the 4 minute trial (>45 seconds), the trial was stopped and the conspecific mouse was removed. As the testing order for the two social investigation tasks may have confounded the results, the social investigation task using juvenile conspecifics was repeated in a separate cohort of mice (n = 5–9 per genotype, per sex). For each social task a different conspecifics mouse was used, so no test mouse was exposed to the same conspecific mouse more than once. All conspecifics were singly housed in a separate room to the test mice one week before social behaviour testing began.

#### Grooming behaviours

Grooming was investigated in the mice during a 10 minute trial, essentially as described [21], however grooming behaviours were recorded by the investigator blind to the genotype group using the Ethovision software.

#### Nesting behaviours

On day 1, mice were placed in a fresh home cage with 60 g of standard food and 90 g of sawdust. 20 g of nesting material was placed in the food hopper on top of the cage. The amount of nesting material left on the food hopper and pulled into the cage was measured 24 hours later. In addition, the dimensions (cm) and weight (g) of the nest were measured.

#### Buried food task

This was performed essentially as described previously [33], except that small chocolate cookies (Nestle Cookie Crisp®, Welwyn Garden City, U.K.) were used as the palatable food.

#### Statistical analysis

All statistical analysis was conducted using Statistica software (Version 5.5, StatSoft, Inc., Tulsa, OK). Data was analysed using either a Student’s t-test, a 2-way ANOVA or a 2-way repeated measures ANOVA, as appropriate. The between-factors were always sex and genotype, and within-factors were either time (home cage), chamber (three-chamber social approach task) or sessions (Morris water maze, DMP). An analysis of covariance (ANCOVA) was used to look at the relationship between activity and anxiety measures, as well as any body weight differences between groups.

## Results

### Reduced Locomotor Activity and Anxiety-like Behaviours

**Figure 1:**
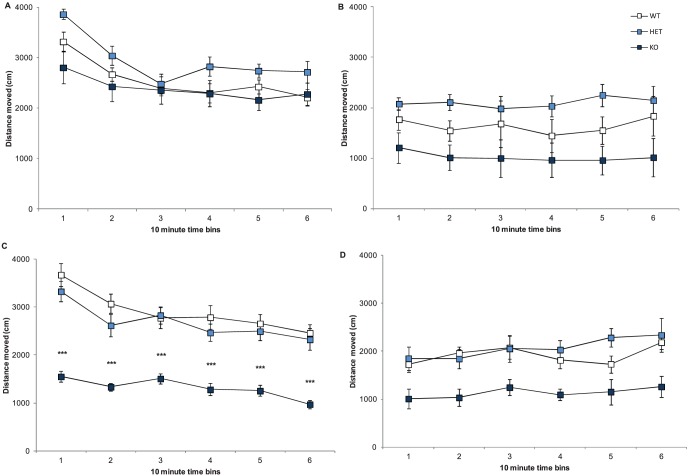
Deletion of Nrxn1α affects locomotor activity in females. Data shown are means (± sem) distance travelled by the Nrxn1α mice during the 3 one hour recordings in the homecage task. Data derived from 23 WT (12M, 11F) 29 HET (15M, 14F) 18 KO (9M, 9F) mice. Activity is shown for the transfer hour (male A, female C) and dark hour (male B, female D). Data from each hour was split into six 10 min time bins. Levels of significance indicated by *** as p<0.001, compared to WT mice.

To explore whether neurexin 1α affects locomotor activity, we tested Nrxn1α KO mice in the homecage task (28), as other mouse models of autism have revealed alterations in spontaneous activity [34]. Across the transfer hour all mice showed habituation to the home cage arena (session factor: F(5,290) = 30.25, p<0.001). The female Nrxn1α KO mice exhibited significantly reduced locomotor activity across both the transfer (sex x genotype interaction: F(2,58) = 8.61, p<0.001) and dark hour (genotype factor: F(2,46) = 12.4, p<0.001; Figure 1), compared to wildtype (WT) and heterozygote (HET) mice. There were no differences in the locomotor activity levels across the light hour session. Male Nrxn1α KO mice showed a trend towards a decrease in the dark hour, but this did not reach significance. Our results indicate that deletion of only one α-Nrxn gene does not lead to general inactivity, but has a specific effect on reducing locomotor activity in novel situations prior to habituation occurring.

**Figure 2:**
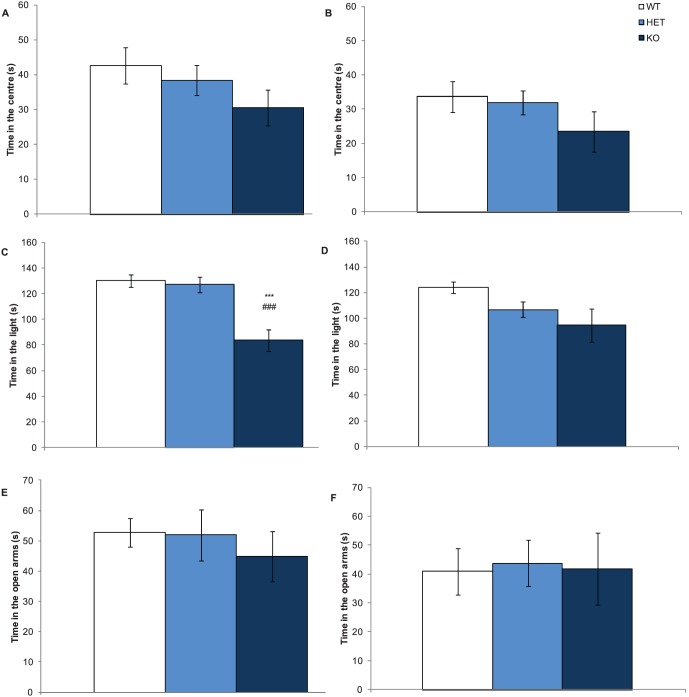
Anxiety is elevated in male Nrxn1α mice. Data shown are anxiety measures taken from the open field, light/dark box and elevated plus maze. Panels are mean (± sem) time spent in the central area of the open field for male (A) and female (B) Nrxn1α mice, time spent in the light compartment of the light/dark box for male (C) and female (D) Nrxn1α mice and time spent in the open arms of the elevated plus maze for male (E) and female (F) Nrxn1α mice. Data derived from 23 WT (12M, 11F) 29 HET (15M, 14F) 18 KO (9M, 9F) mice. Levels of significance indicated by *** as p<0.001, compared to WT mice, and ### as p<0.001, compared to HET mice.

Following investigation of Nrxn1α mice in a habituated (homecage) environment, we assessed the behaviour of the mice in a potentially threatening environment. Anxiety is an associated symptom of autism, occurring in a subset of patients [35], and therefore it is of importance to measure in the Nrxn1α mice. Furthermore, increased anxiety was observed in a mouse model for the neurexin ligand neuroligin 2 [36]. Since Nrxn1α binds to neuroligin 2 [37]–[38], we investigated anxiety-like behaviour by using the open field, light/dark box, and the elevated plus maze. We observed an overall trend for a reduction in the time spent in more anxiogeneic areas of all three anxiety tasks, indicative of increased anxiety in Nrxn1α KO mice (Figure 2). Interestingly, the difference was more apparent in male mice who spent less time in the light compartment of the light/dark box compared to WT and heterozygous mice (genotype factor: F(2,64) = 13.43, p<0.001).

**Figure 3:**
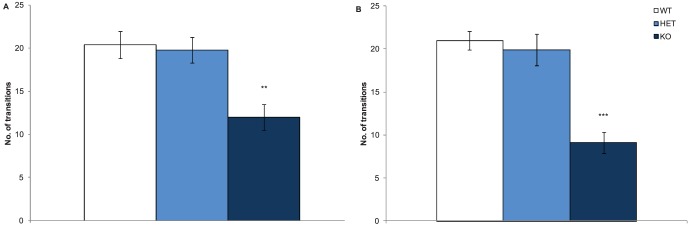
Deletion of Nrxn1α causes reduced locomotor activity in the light/dark box. Data shown are mean (± sem) number of transmissions between the light and dark compartments for male (A) and female (B) Nrxn1α mice. Data is derived from 23 WT (12M, 11F) 29 HET (15M, 14F) 18 KO (9M, 9F) mice, and levels of significance indicated by ** and *** as p<0.1 and p<0.001, respectively, compared to WT mice.

To validate the reduced motor activity observed in the home cage scenario, we also measured the locomotor activity of the mice in the least threatening area of the three anxiety tasks. Reduction in locomotor activity was seen in the light/dark box as all Nrxn1α KO mice made a reduced number of transitions between compartments (genotype factor: F(2,64) = 22.55, p<0.001, see Figure 3) and male Nrxn1α KO mice entered the closed arms of the elevated plus maze significantly fewer times (genotype factor: F (2,63) = 12.74, p<0.001). Analysis of co-variance (ANCOVA) was performed on the results for the activity and anxiety measures in the light/dark box, to assess whether these behaviours were influencing each other in these tasks. The ANCOVA produced a significant interaction effect (F(2,63) = 45.3, p<0.0001), a result which remained significant when co-varying out locomotor activity (F(2,63) = 7.8, p<0.001). However when co-varying out the anxiety measure, the locomotor measure was no longer significant (F(2,63) = 1.24, p = 0.30). These results suggest that an increase in anxiety is the main phenotype in the Nrxn1α KO mice, and that reductions in locomotor activity may be a secondary consequence.

### Nrxn1α KO Mice Show Altered Social Approach

Since impaired social behaviours are a key symptom of ASDs and schizophrenia [39], we investigated social interactions in the Nrxn1α KO mice. Direct social approach in mice has strong face validity to simple social approach behaviours in humans [31], and therefore this is an important behavioural domain to investigate in Nrxn1α deficient mice.

During the 3 trials of the three-chamber social approach task, 3 different behaviours of the Nrxn1α KO mice were tested: locomotor activity, preference for a social cue versus novel object (social approach or sociability) and preference for social novelty. During trial 1, side preference was measured to rule it out as a confounder and no side preference was observed (chamber factor: F(1,67) = 0.001, p = 0.97). Nrxn1α KO mice travelled a significantly shorter distance compared to WT and HET mice in the three-chamber social approach task (genotype factor: F(2,64) = 48.42, p<0.001, see Figure S1).

**Figure 4:**
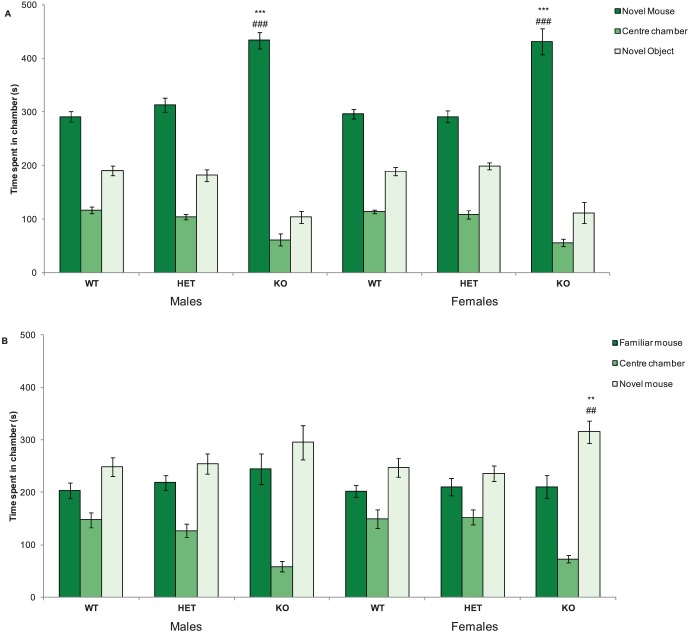
Deletion of Nrxn1α affects the social approach behaviours in mice. Data shown are social approach behaviours of the Nrxn1α mice during the three-chamber social approach task. A – Trial 1 - Mean (± sem) time spent (s) in the chamber containing the mouse, the centre chamber or the chamber containing the object. B – Trial 2 - Mean (± sem) time spent (s) in the chamber containing the familiar mouse, the centre chamber or the chamber containing the novel mouse. Data is derived from 23 WT (12M, 11F) 29 HET (15M, 14F) 18 KO (9M, 9F) mice, and levels of significance indicated by ** and *** as p<0.01 and p<0.001, respectively, compared to WT mice, and ## and ### as p<0.01 and p<0.001, respectively, compared to HET mice.

In Trial 2, social approach, defined as the time the mice spent with either a novel conspecific mouse or novel object, was recorded, with a preference for the novel conspecific mouse indicating social approach or sociability. All mice showed social approach behaviour as all mice spent a greater amount of time in the chamber with the mouse compared to the novel object (chamber factor: F(1,64) = 329.1, p<0.0001), see Figure 4A. However, Nrxn1α KO mice spent a significantly greater ratio of time in the chamber with the mouse versus the object, compared to both the WT and HET mice, suggesting that the Nrxn1α KO mice displayed a greater degree of social approach (genotype factor: F(2,64) = 32.85, p<0.001). Nrxn1α KO mice also spent more time sniffing the wire cup containing the mouse compared to the object than either the HET and WT mice (genotype factor: F(2,64) = 48.96, p<0.001).

Preference for social novelty was also investigated, although this is not thought to be as relevant to autism-like symptoms as social approach as preference for social novelty involves social recognition and social memory [35]. During Trial 3, the female Nrxn1α KO mice spent a significantly greater amount of time in the chamber containing the novel mouse, compared to female WT and HET mice (genotype factor: F(2,64) = 17.92, p<0.001, Figure 4B), suggesting that female Nrxn1α KO mice have a greater preference for social novelty. There was no significant preference for social novelty in any of the male genotype groups. However, preference for social novelty was not observed in the WT mice, suggesting that this task was not sensitive enough to detect social novelty in control mice and therefore results from this trial should be interpreted with caution.

ANCOVA was used to check that changes in locomotor activity were not affecting the social results in trials 2 and 3. Here, both significant genotype effects on social behaviours remained after co-varying activity out (Trial 2 - F(2,62) = 44.7, p<0.001; Trial 3 - F(2,63) = 3.13, p = 0.05). Overall, these results show that deletion of Nrxn1α leads to alterations in social behaviours, a key symptom category in autism and schizophrenia.

### Male Nrxn1α KO Mice Showed Increased Aggression in the Social Investigation Task

To further investigate the social behaviours of the Nrxn1α KO mice, and in particular to access their direct social contact with other mice, social investigation of adult and juvenile conspecifics was measured. In the social investigation task with adult conspecifics, the male Nrxn1α KO mice spent significantly more time in aggressive behaviour compared to WT and HET male mice (Genotype factor: F(2,31) = 10.09, p<0.001). There was also a significant sex effect for time spent being aggressive (Sex effect: F(1,62) = 17.4, p<0.0001), as all female mice did not display any aggression. Furthermore, male Nrxn1α KO mice also spent a greater time social sniffing the conspecifics (Genotype factor: F(2,31) = 5.11, p = 0.01). There were no differences in social investigation in the female Nrxn1α mice.

**Figure 5:**
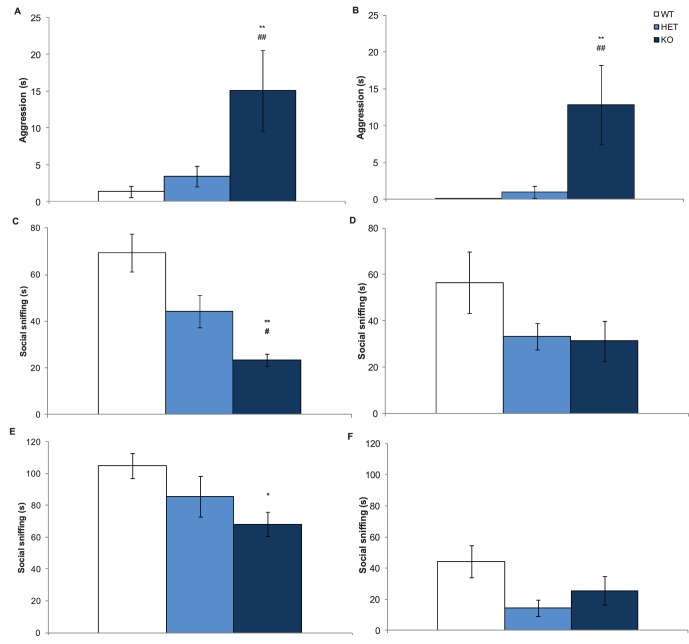
Male Nrxn1α mice show increased aggressive behaviours and social investigation towards a juvenile conspecific. Data shown are the mean (± sem) social investigation behaviours of the Nrxn1α mice. A - Time spent (s) carrying out aggressive behaviours towards the juvenile conspecific by the male Nrxn1α mice in the main study. B - Time spent (s) carrying out aggressive behaviours towards the juvenile conspecific by the male Nrxn1α mice in the replication study. Time spent (s) carrying out social sniffing of the juvenile conspecific mice by the male (C) and female (E) Nrxn1α mice in the main study. Time spent (s) carrying out social sniffing of the juvenile conspecific mice by the male (D) and female (F) Nrxn1α mice in the replication study. Data is derived from 23 WT (12M, 11F) 29 HET (15M, 14F) 18 KO (9M, 9F) mice for the main study, and 16 WT (7M, 9F) 12 HET (6M, 6F) 13 KO (8M, 5F) mice in the replication study, and levels of significance indicated by * and ** as p<0.05 and p<0.01, respectively, compared to WT mice, and # and ## as p<0.05 and p<0.01, respectively, compared to HET mice.

As the Nrxn1α KO male mice show high levels of aggression towards adult conspecifics, we wanted to investigate their social and aggressive behaviours further using juvenile conspecific mice. Juveniles should trigger less aggression in adults [32], permitting assessment of the social behaviour of Nrxn1α KO mice in a less aggressive environment. All Nrxn1α KO mice and male Nrxn1α HET mice spent a significantly reduced time in social investigation with the juvenile conspecific, compared to WT mice (genotype factor: F(2,66) = 7.61, p<0.01; see Figure 5C and E). Due to high levels of aggression observed in male Nrxn1α KO mice towards the juvenile conspecifics (mean [± sem] percentage of time: WT = 1.4(±0.8); HET = 3.4(±1.4); KO = 15.1(±5.5)), the social investigation task had to terminated early in some mice. The Nrxn1α KO male mice spent a significantly shorter time in the trial, compared to both Nrxn1α WT and HET male mice (genotype factor: F(2,33) = 7.08, p<0.01). Male Nrxn1α KO mice also displayed a significantly greater amount of aggression, compared to the Nrxn1α WT and HET male mice (genotype factor: F(2,66) = 5.72, p<0.01; see Figure 5A). This demonstrates that male Nrxn1α KO mice even demonstrated heightened aggressive behaviours towards less threatening juvenile conspecific mice, suggesting a presence of a strong aggressive phenotype in these mice.

To check that the test order for the social investigation tasks (response to adult and then juvenile conspecifics) did not confound the results, this task was repeated in a separate cohort of test mice only exposed to juvenile conspecific mice. In this experiment, male Nrxn1α KO mice again displayed significantly greater number of bouts of aggressive behaviours (genotype factor – F(2,18) = 4.44, p<0.05) and spent significant longer time in aggressive behaviours in response to juvenile conspecifics, compared to WT mice (genotype factor – F(2,18) = 4.18, p<0.05; see Figure 5B). Furthermore, Nrxn1α KO mice spent significantly less time social sniffing juvenile conspecifics (genotype – F(2,35) = 4.41, p<0.05; see Figure 5D and F). There was also a significant sex effect for time spent in anogenital sniffing with males spending more time in anogenital sniffing than females (sex effect – F(1,35) = 13.8, p<0.001). The only apparent effect of conspecific test order appeared to be an increase in social sniffing behaviour displayed by female test mice exposed to adult before juvenile conspecific mice, see Figure 5E and 5F.

Olfactory information is essential for a wide range of mouse behaviours, including social interactions [40], and these social tasks used in the present study depend on olfactory cues. In the buried food task, there were no significant effects of genotype on the time taken to find the food (genotype factor: F(2,64) = 0.01, p = 0.99), however there was a significant sex effect (sex factor: F91,64) = 9.6, p<0.01) with female mice taking longer to find the buried food. Therefore, all mice have an intact sense of smell (see Table S1 in Materials S1), and this is not impacting on their social behaviours.

### Nrxn1α KO Mice Display Reduced Nest Building

**Figure 6:**
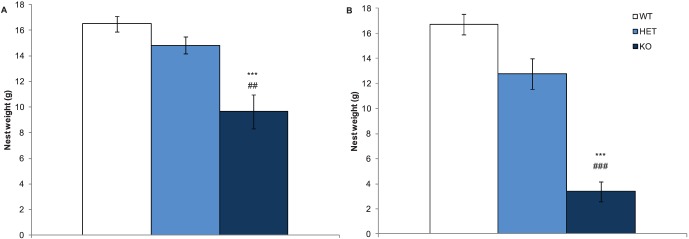
Impairment in nest building behaviours in the Nrxn1α KO mouse. Data shown are the mean (± sem) weight (g) of the nests built by the mice over a 24 hour period. Data is derived from 23 WT (12M, 11F) 29 HET (15M, 14F) 18 KO (9M, 9F) mice, and levels of significance indicated by *** as p<0.0001, compared to WT mice, and ## and ### as p<0.01 and p<0.001, respectively, compared to HET mice.

Nest building is considered a normal home cage behaviour [41] and has previously been associated with maternal care [42]. As a significant reduction in nest building was found in Nrxn1α KO mice maintained on a mixed genetic background [21], this test was repeated in the current study. All Nrxn1α KO mice displayed reduced nest building, compared to WT and HET mice (see Figure 6). The nest weight (genotype factor: F(2,64) = 51.86 p<0.0001), nest width (genotype factor: F(2,64) = 3.37 p<0.05) and nest length (genotype factor: F(2,64) = 4.05 p<0.05) were reduced in Nrxn1α KO mice. There was also a significant sex effect for nest weight (sex factor: F(1,64) = 11.78, p<0.01) with female mice making smaller nests. Overall, this shows that the impairment in nest building behaviours is a strong phenotype that is present in both the backcrossed Nrxn1α KO mouse and mutants maintained on a mixed background.

### Nrxn1α KO Mice Show no Impairments in Spatial or Working/Episodic Memory

**Figure 7:**
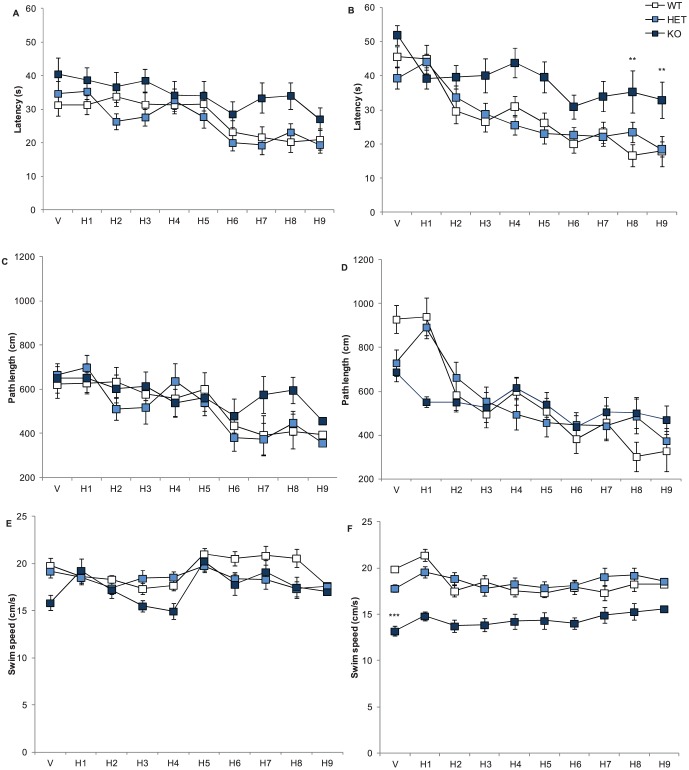
Nrxn1α mice show no impairment in spatial working. Data shown are mean (± sem) behaviours of the Nrxn1α mice during the Morris water maze. Latency to reach the platform for male (A) and female (B) mice, path length for male (C) and female (D) mice, and swim speed for male (E) and female (F) mice. Data is derived from 23 WT (12M, 11F) 29 HET (15M, 14F) 18 KO (9M, 9F) mice, and levels of significance indicated by ** and *** as p<0.01 and p<0.0001, respectably, compared to WT mice.

Since cognitive deficits, including impairments in spatial memory, are commonly seen in schizophrenia [43] and some ASD patients with neurexin mutations have been found to have low IQs [44], Nrxn1α KO mice were tested in the Morris water maze task. Mice underwent 4 trials per day, across a 10-day period. Across the test sessions, all mice showed a significant reduction in the latency to find the platform (session factor: F(8,512) = 17.49, p<0.0001, see Figure 7). There was no effect of Nrxn1α genotype on performance in the Morris water maze task in male mice. Female Nrxn1α KO mice had significantly increased latency to reach the platform across the task (genotype factor: F(2,64) = 18.3, p<0.0001), compared to female WT and HET mice. However, female Nrxn1α KO mice also had significantly reduced swim speeds across the task, compared to WT and HET mice (genotype factor: F(2,64) = 27.62, p<0.001; Figure 7). The reduction in swim speed in the female Nrxn1α KO mice appeared to be driving the observed increase in latency to reach the platform, suggesting that there was not a spatial memory impairment in the female Nrxn1α KO mice, just a reduction in locomotor activity. Furthermore, there was a significant reduction in path length across the ten days in both male and female mice (session factor: F(8,512) = 14.78, p<0.0001) which also indicates that there was no effect of Nrxn1α KO on spatial learning in either sex.

**Figure 8:**
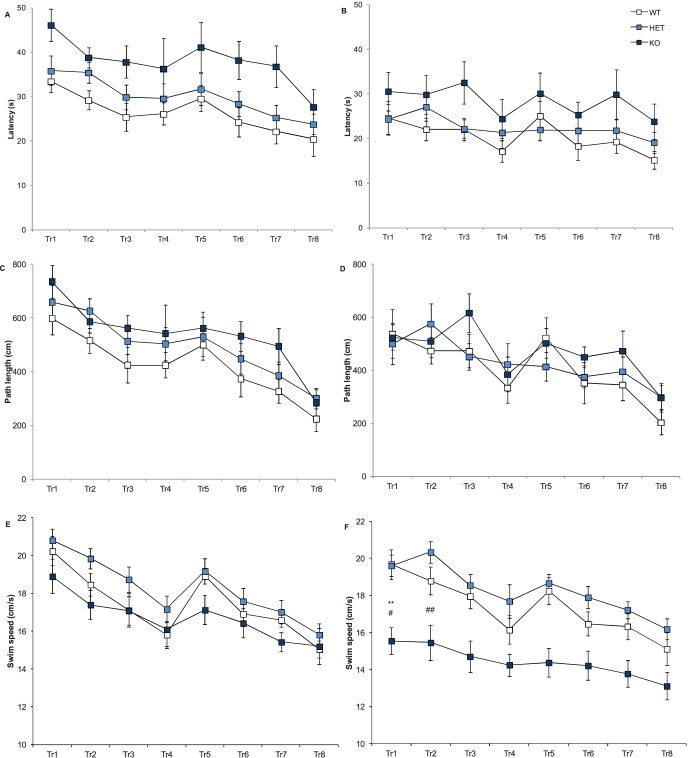
Nrxn1α mice show no impairment in working/episodic memory. Data shown are mean (± sem) behaviours of the Nrxn1α mice during the delayed matching-to-place task. Latency to reach the platform for male (A) and female (B) mice, path length for male (C) and female (D) mice and swim speed for male (E) and female (F) mice. Data is derived from 23 WT (12M, 11F) 29 HET (15M, 14F) 18 KO (9M, 9F) mice, and levels of significance indicated by * as p<0.05, compared to WT mice, and # as p<0.05, compared to HET mice.

Because patients with schizophrenia exhibit deficits in a range of cognitive functions, including working and episodic memory, we used a delayed matching-to-place (DMP) task to assess working/episodic-like performance in the Morris water maze. A reduction in the time taken to find the platform between trial 1 and trial 4 during each test session (‘saving time’) is taken as a measure of working/episodic-like memory. Mice displayed a ‘saving time’ in their latency to reach the platform over the 4 test sessions (session factor: F(3,192) = 7.32, p<0.001) however there were significant sex and genotype effects (sex factor: F(1,64) = 9.7, p<0.05; genotype factor: F(2,64) = 10.4, p<0001), as the Nrxn1α KO mice did take longer to reach the platform and in general females were quicker to reach the platform than males (Figure 8). All mice also significantly reduced their path length throughout the 4 sessions (session factor: F(3,192) = 8.14, p<0.001), and there was a significant sex effect as females had on average a shorter path length than males (sex factor – F(1,64) = 4.42, p = 0.04). For swim speed, there is again a significant session factor across the 4 sessions (session factor: F(3,192) = 32.1, p<0.0001) as all mice reduce their swim speed, however the Nrxn1α KO mice show significantly reduced swim speed compared to WT and HET mice (genotype factor: F(2,64) = 12.9, p<000.1; Figure 8). As with the standard Morris water maze, the Nrxn1α KO mice had significantly reduced locomotor activity in the DMP, leading to the observed increase in latencies in the Nrxn1α KO mice confounding the assessment of working/episodic-like memory using latency measures in this task. However, the lack of genotype effect on the path length measures in this task, further support a lack of effect of Nrxn1α deletion on working/episodic-like memory.

### Nrxn1α KO Mice Show no Impairments in Short or Long Term Memory or Repetitive Behaviours

Cognitive deficits have been seen across a variety of neurodevelopmental disorders [45]–[46] and therefore working memory in Nrxn1α KO mice was assessed. The novel object task was carried out where mice are exposed to a series of novel and familiar objects to access both short-term and long-term memory, however no deficit in working memory was identified (see Table S1 in Materials S1).

Another core feature of autism is repetitive/stereotyped patterns of behaviour [39], and increased grooming has been previously found in Nrxn1α KO mice maintain on a mixed background [21]. However, no difference in grooming behaviours in the Nrxn1α KO mice was seen in the present study (see Table S1 in Materials S1).

## Discussion

We hypothesized that deletion of the Nrxn1α gene in a pure genetic background would lead to alterations in behaviours relevant to behavioural abnormalities seen in humans with NRXN1 genetic deletions. Furthermore, electrophysiological recordings in central and peripheral nervous systems revealed that genetic deletion of Nrxn1α in mice reduces spontaneous and evoked release [4], [8], [21]. Since exonic deletions in the NRXN1 gene in human patients were linked to autism and schizophrenia [15]–[16], we wanted to test whether the impairment in transmission may also lead to behavioural abnormalities related to the diseases. In this study we have shown that homozygous deletion of the Nrxn1α gene maintained on a single genetic background resulted in increased social approach, a reduction in social investigation towards a juvenile conspecific and reduced locomotor activity in novel environments. In addition, male KO mice exhibited an increase in aggressive behaviour towards both juvenile and adult conspecifics. These results suggest that Nrxn1α may play a role in regulating locomotor activity and social behaviour in mice. This is the first report linking a deletion in Nrxn1α to alterations in social behaviour, one of the core symptom domains affected in ASDs, and therefore our findings suggest that deletions within the NRXN1 gene found in patients may be responsible for the impairments seen in social behaviours.

### Altered Social Behaviours in the Nrxn1α KO Mouse

In the present study the most significant finding is alterations in social behaviours. There are a number of ways in which Nrxn1α KO mice may show increased social approach in the three-chamber social approach task. Firstly, deletion of Nrxn1α may interfere with the encoding of social information and therefore it may take the knockout mice longer to interpret social cues given off by the conspecific mouse, causing them to spend a greater amount of time investigating them. Alternatively, as the Nrxn1α KO male mice also show a significantly increased level of aggression, it may be this that is causing them to appear to have greater social approach. Differences in aggression between male and female mice is commonly reported [47]–[49] with males tending to show higher levels of aggression towards intruders throughout their adult life, however females only show fierce aggression to intruders during lactation and rearing pups [49]. This may explain why aggression was not observed in female Nrxn1α KO mice. As the mice were singly housed one week before testing began (and 2 months before the social investigation tasks occurred) this may have heightened the aggressive behaviours. However the aggressive phenotype was observed in the replication study in which the test mice had only been singly housed for one week prior to testing so the increased aggression observed in the male Nrxn1α KO mice is probably not a results of extended single housing. During the two social investigation tasks, the male Nrxn1α KO mice show different patterns of social sniffing: they displayed an increase in social sniffing towards an adult conspecific but a decrease when paired with a juvenile conspecific. In the replication cohort, the social investigation task was only performed using juvenile conspecifics to examine whether test order had confounded the results. As the reduced social sniffing of the juvenile conspecific was replicated, this indicates that the order of testing did not affect the behavioural results, and that reduced social investigation is a robust phenotype of Nrxn1α KO mice. Although it is interesting that Nrxn1α KO male mice showed different social behaviour towards conspecific mice of different ages, further experiments would need to be conducted to determine why the social phenotype of these mice depends on the age of the conspecific and whether this altered social behaviour is present early in development in Nrxn1α KO mice.

In this study we have also shown that Nrxn1α KO mice have a significant reduction in nest building behaviours, a phenotype that was also observed in Nrxn1α KO mice maintained on a mixed background [21]. Previous research has shown that nesting helps to facilitate family structure and maternal interaction [50]. Although pregnant females tend to build the most complex nests, nesting behaviour is also relevant to male mice and non-pregnant females, where impairments in nest building can provide early indications of aberrant social behaviours [41],[51]–[52]. In addition to the Nrxn1α KO mice, changes in nesting have been associated with abnormalities in social behaviour observed in other mouse models. For example, Dvl1 mutants, which provide a model of several human psychiatric disorders, show impairment in nesting building behaviours as well as reduced social interactions and abnormal PPI [41]. Furthermore, mice lacking the Schurri-2 gene, a major histocompatibility complex enhancer gene, have been found to have schizophrenia-related behavioural abnormalities, including impairment in nest building [53]. These findings, as well as the fact that administration of psychomimetic agents has been shown to disrupt nest building in mice [54], further suggest that nest building impairments in mice may be associated with social behaviour of relevance to psychiatric disorders.

### Previous Behavioural Findings of the Nrxn1α KO Mouse

In addition to the social behavioural findings, there are a few inconsistences between this study and previous behavioural results. An increase in repetitive grooming behaviours has previously been reported [21], and an increased response to novelty, measured by locomotor activity reduction over 3 trials, were found in male Nrxn1α mice [22]. However we found the opposite, where all Nrxn1α mice show a reduction in locomotor activity in novel situations. Both previous reports also tested the mice on numerous social tasks, however did not find any significant alterations in social behaviours. In this study, the research was carried out on mice that had been backcrossed onto a single genetic background (C57BL/6J), as opposed to previous behavioural analysis of the mice that were maintained on a mixed genetic background (C57BL6/SV129) which could result in individual genetic variation at, or separate to, the targeted locus that might confound or obscure the behavioural analysis [21]–[22]. The Nrxn1α KO mouse was engineered in embryonic stem cells from the SV129 sub-strain of mice, because these cell lines are amenable to targeting by homologous recombination. A congenic line made with unrelated strains is statistically expected to be roughly 97% from the recipient after 5 generations of backcrossing [55], with each round of backcrossing causing host genome sequences to be lost exponentially, thus decreasing the congenic footprint flanking the targeted mutation. Prior to backcrossing, this congenic footprint will contain numerous genes, which may contribute to the behavioural phenotype, making any interpretation difficult. It has previously been shown that, for example, PPI is enhanced in Fmr1 (fragile X mental retardation 1 gene) KO mice on a pure C57BL/6J background, but normal when the mutants are maintained on a mixed C57BL/6J x FVB/NJ background [56], highlighting the importance of transferring the allele onto a single genetic background. Furthermore, a mixed background could also contribute to genetic variation at regions outside the targeted locus, seriously confounding phenotypic assessment [26].

### Animal Models of Neurodevelopmental Disorders

The development of reliable, predictive animal models for the traits related to complex disorders, such as autism and schizophrenia, is essential to increase our understanding of the disorders and develop disease models that can be used in the development of new treatments. This study was designed to investigate the role of Nrxn1α in a variety of mouse behaviours potentially analogous to autism and schizophrenia, and not to directly mimic the deficits associated with the deletion in humans. A fundamental issue surrounding mouse models of neurodevelopmental disorders is translatability, i.e. the ability to identify which specific behaviours in the mouse reasonably correlate with the symptom categories for each disorder, as well as the reproducibility of such models. The relationship between animal and human behaviour, especially in relation to genotype-phenotype correlation, is not clear, however there is a body of research aimed at generating translatable rodent tests which simply focus on core symptoms, such as abnormal social interaction and repetitive behaviours for autism, and working and spatial memory tasks for schizophrenia [57]–[58].

In addition, there are a number of important considerations for cross-species validity of mutations [59]–[60], especially CNVs. In rodents, full knockouts are usually studied but in human disease copy number mutations are rarely knockouts, but hemizygous deletions or duplications of highly dosage-sensitive genes. This allows for the function of the gene to be explored in the mouse model, however translation back to the human hemizygote CNV is not usually so straightforward. Secondly, the phenotypes observed in animals may be less deleterious than those seen in humans. This may be due to differences in gene function between the species or changes in compensatory mechanisms, but highlights why you may see phenotypes in a KO mouse and not a HET mouse. In the present study, a lack of HET effect may be due to compensatory actions of other transcripts, such as the β-isoform which is still intact in these mice, unknown alternative NRXN1 transcripts, the Nrxn2 and/or Nrxn3 genes, or functional differences between the human and mouse Nrxn genes.

One notable feature of NRXN1 deletions, as for many rare pathogenic largely *de novo* CNVs [61]–[64], such as deletion of 15q13.3 [65]–[66], 16p11.2 [63], [67] and 1q21.1 [66], [68], is their association with a range of neurodevelopmental disorders including autism, schizophrenia, epilepsy and intellectual disability, i.e. they have pleiotropic effects [69]–[70]. In addition, genetic variation within NRXN1 has been shown to confer neural and cognitive susceptibility common to both schizophrenia and ASDs, providing one possible mechanistic explanation for the pleiotropic effects in the brain [70]. Therefore, the social dysfunction observed in the Nrxn1α KO mice does not relate to a specific neurodevelopmental disorder.

In conclusion, we have provided the first evidence that deletion of the Nrxn1 gene in mice leads to alterations in social behaviours, as well as locomotor activity, anxiety, normal home cage behaviours and aggression. Due to the link between NRXN1, ASDs and schizophrenia in humans, these results are compelling. As ASDs and schizophrenia are thought to be multigenic disorders, our findings suggest that deletions within the NRXN1 gene found in patients may be associated with the impairments seen in social behaviours.

## Supporting Information

Figure S1 Deletion of Nrxn1α causes reduced locomotor activity. Data shown are mean (± sem) distance moved (cm) during trial 1 of the three-chamber social approach task for male (A) and female (B) Nrxn1α mice. Data is derived from 23 WT (12♂, 11♀) 29 HET (15♂, 14♀) 18 KO (9♂, 9♀) mice, and levels of significance indicated by *** as p<0.001, compared to WT mice, and ### as p<0.001, compared to HET mice.
(EPS)

Materials S1 Supplemental Materials and Table S1, results from the novel object recognition, grooming and buried food tasks for the Nrxn1α WT, HET and KO mice. Values are mean (± sem) for the time spent exploring the two objects for trials 1 and 2, and for both short-term memory (STM) and long-term memory (LTM) in the object recognition task, grooming and rearing behaviours in the grooming task, and latency to find the hidden food in the buried food task. Data is derived from 23 WT (12**♂**, 11**♀**) 29 HET (15**♂**, 14**♀**) 18 KO (9**♂**, 9**♀**) mice, and levels of significance indicated by ** and *** as p<0.01 and p<0001, respectively, compared to WT mice.
(DOCX)
