# Supplementary material for: Altered Social Behaviours in Neurexin 1α Knockout Mice Resemble Core Symptoms in Neurodevelopmental Disorders
Source: PLoS One. 2013 Jun 28;8(6):e67114. doi: 10.1371/journal.pone.0067114 (PMC3696036; doi:10.1371/journal.pone.0067114)
Supplement: Materials S1 — Supplemental Materials and Table S1, results from the novel object recognition, grooming and buried food tasks for the Nrxn1α WT, HET and KO mice. Values are mean (± sem) for the time spent exploring the two objects for trials 1 and 2, and for both short-term memory (STM) and long-term memory (LTM) in the object recognition task, grooming and rearing behaviours in the grooming task, and latency to find the hidden food in the buried food task. Data is derived from 23 WT (12♂, 11♀) 29 HET (15♂, 14♀) 18 KO (9♂, 9♀) mice, and levels of significance indicated by ** and *** as p<0.01 and p<0001, respectively, compared to WT mice. (DOCX) [file pone.0067114.s002.docx]

**Supplemental information**

**Methods and Materials**

**Mice**

The mice were genotyped by PCR using genomic mouse DNA from ear punches (WT primer: CGA GCC TCC CAA CAG CGG TGG CGG GA, KO primer: GAG CGC GCG CGG CGG AGT TGT TGA C, common primer: CTG ATG GTA CAG GGC AGT AGA GGA CCA). All mice were individually housed one week prior to testing in Tecniplast cages (32cm x 16cm x 14cm) with sawdust (Litaspen premium, Datesand Ltd, Manchester), a cardboard shelter and additional bedding material (Sizzlenest, Datesand Ltd, Manchester) with *ad libitum* access to water and food (Rat & Mouse # 1 Diet, Special Diet Services, Essex, UK). Cages were changed once every two weeks but never on the day before, or the day of, testing to minimize the disruptive effect of cage cleaning on behaviour. The housing room was maintained at constant room temperature (21 °C) and humidity (45%) and kept under a regular light/dark schedule with lights on from 08:00 to 20:00 hours (light = 270 lux). As singularly housing can be classed as a stressor, this was only done when the mice were adults (>10 weeks old) to reduce its effects on behaviour. The oestrous phase of the female mice was not checked in this study. However, it is unlikely that this affected results because there were no major effects in the variance between males and females.

**Behavioural tests**

Tests were carried out in the following order: home cage, open field, light/dark box, elevated plus maze, novel object discrimination, three-chamber social approach task, grooming and nesting behaviour, social investigation with an adult conspecific, Morris water maze, delayed matching-to-place task in the water maze, social investigation with an adult conspecific and food burying test for olfactory ability. After each test, boli and urine were removed from the test arena which was then cleaned with 1% Trigene**®** solution. All behavioural tests were performed in the light phase between 09:00 and 18:00 hours. Experimenters were blind to the genotypes of the animals both during testing and subsequent scoring of the recorded behaviour. The mice were re-genotyped following behavioural testing to confirm genotype.

**Home cage** [1]**:** The spontaneous activity cage was adapted from the standard mouse-housing cage (Tecniplast cage, 32cm x 16cm x 14cm) by moving the food hopper and water bottle to the side of the cage, which gave *ad libitum* access from inside the cage and the metal grid cage lid is replaced with a transparent Plexiglas lid on the top. The test room lighting (250 lux) and cycle matched that of the housing room. Twelve red cluster lights (LED cluster red light No. 310-6757; RS Components Northants, UK) of approximate wavelength 705 nm provided minimal red light for video recording during the dark phase. The home cage task assesses the spontaneous locomotor activity of mice for 3 separate hour periods in a novel environment to which the mice become familiar over a 23 hour period. Mice were placed in the cage for 23 hours and their activity was recorded for one hour immediately after transfer (12.00-13.00; transfer hour), an hour in the middle of their dark phase (01:00-02:00; dark hour) and an hour 22 hours after transfer (11.00-12.00; habituation hour). For the analysis, activity measures were collapsed into six 10 min bins to assess temporal patterns of activity over each hour period.

**Open field** [2]**:** The open field test measures the conflict between rodents’ exploratory behaviour and aversion to open areas. Mice were placed facing the wall of an open field arena (72 x 72 x 33 cm) and allowed to freely explore the arena for 10 min. A small lamp placed on the test room floor provided dispersed lighting (25 lux). Each mouse was placed in the corner of the open field at the beginning of the trial. In the Ethovsion software, a square of equal distance from the periphery (36 x 36cm), defined as the ‘central zone’, was virtually drawn within the arena. The frequency of entries into, and the time spent in, the central zone of the arena (36 × 36 cm) were extracted in Ethovision, in addition to the total distance travelled (cm) and mean velocity (cm/ second).

**Light/dark box** [3]**:** The light dark box measures the conflict between rodents’ exploratory behaviour and aversion to open and brightly lit areas. For the light/dark test, a custom-built box of white acrylic was used with dimensions (44 x 21 x 21 cm). The box was divided into two chambers by a sheet of white acrylic (21 x 50 cm); a smaller dark chamber (20 lux) that occupied roughly 1/3 of the total box, and a larger light chamber (80-110 lux) that was lit from above with a bright white light. A small doorway within the partition, 5 x 7 cm, allowed the mice to move between chambers freely. Mice were placed in the dark compartment at the start of the 5-min trial. The latency (s) to enter the light chamber, activity within the two chambers (cm) and number of light-dark transitions were measured. The mean velocity (cm/s) and total distance travelled (cm) in the dark and light compartments were extracted from the tracking software. As the tracking system is less sensitive at determining a transition between the compartments, particularly when a mouse sits between the compartment partition, the number of entries into, and the time spent in, the light compartment were manually scored. Entry to either compartment was defined as when all four paws of the mouse had entered in one compartment.

**Elevated plus maze** [4]**:** The elevated plus maze measures the conflict between rodents’ exploratory behaviour and aversion to open and elevate areas. The apparatus was made of perspex and consisted of two opposing closed arms (30 x 5 cm), which were enclosed by 15 cm high clear perspex walls, and two opposing open runways (30 x 5 cm), with 0.5 cm high clear perspex ledges around the open arms. The maze was elevated 40 cm form the ground on a transparent acrylic stand. Light intensity in the open arms was 40 lux, compared to 20 lux in the closed arms. Mice were placed on the central platform, facing one of the closed arms, and left to explore the maze undisturbed for 5 min. The number of entries into, and the time spent in, the open and closed arms were manually scored. Locomotor activity measures of velocity (cm/s) and distance travelled (cm) were extracted from the Ethovision software. An entry into an arm was defined as when all four paws of the mouse had entered an arm, and an exit from an arm was defined as when the forepaws of the mouse had left the arm.

The open field test [[5](#_ENREF_5)], the light dark box [[3](#_ENREF_3)] and the elevated plus maze [[4](#_ENREF_4)] were used to measure anxiety-like behaviours in the mice. Each test measures the conflict between rodents’ exploratory behaviour and aversion to open and brightly lit areas. Decreasing entries and time spent in the anxiogenic areas is indicative of increased anxiety-like behaviour and is reversed by treatment with anxiolyics and antidepressants [[2](#_ENREF_2)].

**Novel object discrimination** [6]-[7]**:** Based on the tendency of mice to approach and explore novel objects, discrimination of novel objects across time-separated trials provides an indirect measure of short and long-term memory. The task is carried out in an empty home cage (Tecniplast cage, 32cm x 16cm x 14cm) and mice are exposed to different object combinations, black or white cubes or pyramids, over four 10 min trials to measure the exploratory activity of the mouse in response to a novel object placed within the home cage. Low lighting (30 lux), was evenly distributed across the arena during testing. During the first familiarisation session, mice are allowed to freely explore two identical objects for ten min. One hour later, the same procedure was carried out but one object was changed to introduce a different (novel) object (discrimination session – short term memory). The following day, the animals were again exposed to two identical objects, different to the ones previously seen in the first familiarisation session. 24 hours later (long term memory), two objects were placed into the home cage, one familiar object from the previous day (familiarisation session 2) and one novel object, and then the mice were left to explore the two objects for ten min (discrimination session – long term memory). The type and location of the objects were counterbalanced to minimise any potential confound due to a preference for object type and/or location. Latency (s) to initially explore the objects, as well as the frequency of, and time (s) spent in, object exploration, and rearing behaviours during each 10 min trial were measured.

**Morris water maze** [8]**:** This is a standard and commonly used spatial navigation task that requires mice to swim to a submerged platform (target) by means of triangulation using navigational cues [[8](#_ENREF_8)]-[[9](#_ENREF_9)]. The water maze was a white circular pool (1.3 m inner diameter and walls 60 cm high), filled with tap water (r21°C) up to 30 cm deep. Although there is no standard size for the pool used in the Morris water maze task [[10](#_ENREF_10)], a 1.3 m diameter pool should be sufficient to allow reliable estimates of performance but not so large as to be aversive [[11](#_ENREF_11)]. A non-toxic, white aqueous emulsion (Acusol OP301 Opacifier, Rohm & Haas, Landskrona, Sweden) was used to make the water opaque to prevent the mice visualising the platform in the hidden platform sessions and to aid tracking of the mice in the pool by the Ethovision software. The pool was lit from below with white light (100 lux) and surrounded by cream-coloured curtains from which a set of extra-maze cues were suspended to help the mice navigate around the pool. Four equidistant positions around the edge of the pool were designated as Target (T), Opposite (O), Left (L) and Right (R), dividing the arena into equivalently sized quadrants. The quadrants served as alternate start locations for the successive trials which were run in a pseudorandom manner. The platform had a round surface (10 cm diameter) and was positioned just above the water level for the visual platform trials on day 1, and 1 cm under the water level for the following 9 days of hidden platform trials. The mice were run in squads of 6 mice/squad, and each mouse underwent four trials per day (i.e one session a day). For each session, mice were taken into the test room in squads and a new trial started when all 6 mice had finished the previous trials. Between trials, mice were returned to their home cages and at the end of each session of 4 trials, mice were returned to the housing room. On the first day the mice were trained to locate the visible platform in the pool. The trials were started by placing the mouse into the pool close to, and facing, the pool wall in one of the four start locations (each subsequent trial was started in a different quadrant in a pseudorandom order). Each mouse was given 60s to swim to the platform and allowed to remain on the platform for 30 s before being removed by the experimenter. The latency to reach the platform was recorded from when the mouse was put into the pool to when all four paws of the mouse were on the platform. If the mouse did not reach the platform after 60 s it was guide to the platform. Faecal boli were removed between each trial and the water changed weekly. The same protocol was used over the subsequent 9 days but on these daily sessions, the platform was hidden below the water level On the final day (Day 10), a probe task was run in which the platform was removed and the mice allowed to swim in the pool for 60 s. To assess the retention of spatial memory, the time spent in the quadrant that had contained the platform (target quadrant) compared to the other quadrants was measured. Latency to reach the platform was manually scored for each mouse by an experiment blind to the genotype of the mouse and path length (cm) to reach the platform and swim speed (cm/s) were extracted from the Ethovision software. Mean latencies (s) and path lengths (cm) were calculated across the trials within each session for each mouse. Conflicting behavioural responses such as floating or thigmotaxis (the amount of time spent swimming in the outer area of the pool defined as a 15 cm wide circular zone adjacent to the wall of the maze) were assessed throughout the trials.

**Delayed matching-to-place** [12]-[13]**:** The delayed matching-to-place task (DMP) version of the Morris water maze has been proposed as a model of working/episodic-like memory and has been shown to involve the hippocampus and N-methyl-D-aspartate receptor activation [[13](#_ENREF_13)]-[[14](#_ENREF_14)]. The protocol was as described above except that mice underwent 8 trials/day for 7 days and the platform location was changed each day (given the increased difficulty of the DMP task, mice were allowed a maximum of 90 s to find the platform). Latency to reach the platform was manually scored for each mouse by an experimenter blind to the genotype of the mouse and path length (cm) to reach the platform and swim speed (cm/s) were extracted from the Ethovision software. Mean latencies (s) and path lengths (cm) were calculated for each trial across the 7 sessions for each mouse. The reduction in latency to find the platform in subsequent trials compared with the first trial within a session reflects the ability of the mouse to rapidly acquire memory for the new platform location, based on the experience gained in the first trial, against the interference from the memories of previous platform locations. The reduction in latencies to find the platform between the first and subsequent trials is referred to as ‘saving time’ and is used as an index of working/episodic-like memory.

**Three-chamber social approach task** [15]**:** A major symptom category of autism is unusual reciprocal social investigations, and direct social approach in mice has strong face validity to simple social approach behaviours in humans, which are frequently impaired in autism. The three-chambered social approach task monitors direct social approach behaviours when a subject mouse is presented with the choice of spending time with either a novel mouse or novel object. Sociability is defined as the subject mouse spending more time in the chamber containing the mouse than in the chamber containing the object. A subsequent 10-min trial was performed to measure preference for social novelty, although this is not thought to be as relevant to autism-like symptoms as sociability. In this trial, the subject mouse is presented with the choice of either the familiar mouse from the previous trial, or a novel mouse. Preference for social novelty is defined as spending more time in the chamber with the novel mouse than in the chamber containing the familiar mouse. The apparatus is a rectangular three-chamber box, where each chamber measures 20 cm (length) x 40.5 cm (width) x 22 cm (height). Dividing walls are made from clear perplex, with small openings (10 cm width x 5 cm height) that allow access into each chamber. The task was performed as described previously [[15](#_ENREF_15)] except that the conspecific mice were habituated in the wire cups for 15 min prior to undergoing a trial in the three-chamber arena. The three chamber task was lit from below (10 lux). The mice were allowed to freely explore the three-chamber apparatus over three 10 min trials. During trial 1, the apparatus was empty and the locomotor activity (distance travelled, cm; velocity, cm/s) of, and time (s) spent in each chamber by, the mice was tracked using the Ethovision software. In trial 2, one wire cup was placed upside down in one of the side chambers (novel object stimulus) and a novel juvenile conspecific mouse was placed under another wire cup in the other side chamber (novel mouse stimulus), leaving the middle chamber empty. The location of the novel mouse across trials was counterbalanced to minimise any potential confound due to a preference for chamber location. During trial 3, a novel juvenile conspecific mouse was placed under the second, empty wire cup. The time spent exploring the object or the conspecific mice were manually scored by an experimenter blind to the genotype of the mouse.

**Social investigation task** [16]**:** Mice were transferred to a clean cage, identical to their normal homecage, (containing only sawdust), 1 hour before testing to habituate. The testing area was lit from below with a low light (10 lux). During testing, Mice were transferred into the testing room in their ‘new’ homecage, a conspecific mouse was put into the cage with them, and their social investigation was scored. The mice were allowed to interact with a freely moving same-sex novel adult (aged 12 weeks) or juvenile (aged 4 weeks) mouse for two separate 4 minute trials. Social behaviours of anogenital sniffing and social sniffing (sniffing any region of the conspecific mouse from the trunk upwards) as well as allogrooming (where the test mouse grooms the conspecific mouse) and aggression were manually scored by an experimenter blind to the genotype of the mouse. When aggression was observed for prolonged periods (>45 seconds), the trial was stopped and the conspecific mouse was removed.

**Grooming behaviour** [17]**:** Each mouse was placed individually in a clean standard home cage **(**Tecniplast cage, 32cm x 16cm x 14cm**),** with no sawdust or nesting material, and allowed to habituate for 10 min under red light (six red cluster lights, see above for details). Following habituation, mice were observed for another 10 min and the time (s) spent self-grooming was manually scored by an experimenter blind to the genotype of the mouse. Locomotor activity (distance travelled, cm; velocity, cm/s) of the mice was tracked using the Ethovision software.

**Nesting behaviour** [17]**:** Nesting behaviour has previously been identified as a normal home cage social activity and an important parental behaviour [[18](#_ENREF_18)]-[[19](#_ENREF_19)]. On day 1, mice were placed in a fresh home cage with 60g of standard food and 90g of sawdust. 20g of nesting material was placed in the food hopper on top of the cage. The amount of nesting material left on the food hopper and pulled into the cage was measured 24 hours later. In addition, the dimensions (cm) and weight (g) of the nest were measured. (Nesting behaviour was assessed in the housing room).

**Buried food task** [20]**:** This was performed essentially as described previously [[20](#_ENREF_20)], except that small chocolate cookies (Nestle Cookie Crisp®, Welwyn Garden City, U.K.) were used as the palatable food. A cookie was placed in the home cage of each mouse for three consecutive days to habituate them to the food before being food deprived for 24 hours prior to testing. On the day of the test, the mice were placed into a clean cage containing sawdust at a depth of 5cm (Litaspen premium, Datesand Ltd, Manchester) and left to habituate in the test room for 5 minutes. (The light light level in the test room was 10 lux). The mice were then removed from the cage while a cookie was hidden 2 cm below the surface of the sawdust at the top of the cage (left, middle or right location, counterbalanced across genotype groups). The mouse was returned to the cage and the latency for the mouse to find the cookie was recorded, as was the amount of cookie eaten within 5 minutes of finding the cookie.

**Results**

**Nrxn1α KO mice show altered social approach**

During trial 1 of the 3 chamber social approach task, Nrxn1α KO mice travelled a significantly shorter distance compared to wildtype and heterozygote mice in the three-chamber social approach task (genotype factor: F(2,64)=48.42, p<0.001, see Figure S1).

**Nrxn1α KO mice show no impairments in long or short term memory, spatial learning and memory, working memory or repetitive behaviours**

Novel object

To determine whether there were working memory deficits in the Nrxn1α KO mice, the novel object task was carried out where mice are exposed to a series of novel and familiar objects to access both short-term and long-term memory. Cognitive deficits have been seen across a variety of neurodevelopmental disorders [[21](#_ENREF_21)]-[[22](#_ENREF_22)] and therefore working memory in Nrxn1α KO mice was assessed. The mice showed an object preference, spending more time exploring the pyramid-shaped objects, compared to the cube-shaped objects (object factor: F(1,64)=23.4, p<0.0001) and also an object location preference during trial 1 (location factor: F(1,64)=6.8, p<0.05). However these preferences should have had a minimal effect as object type and location were counterbalanced across genotype groups. There was no effect of genotype on the time spent exploring the objects or in the discrimination between familiar and novel objects in either the short or long-term memory conditions (STM – genotype factor: F(2,58)=0.23, p=0.79; LTM – genotype factor: F(2,58)=0.12, p=0.89; see Table S1).

Grooming behaviours

Another core feature of autism is repetitive/stereotyped patterns of behaviour [23], and increased grooming was being previously found in Nrxn1α KO mice maintain on a mixed background [[17](#_ENREF_17)]. Therefore, repetitive grooming behaviours were assessed in the present study. There was no significant differences observed in either the amount of time spent in, or number of bouts of grooming during the task. There was a significant increase in rearing behaviour in Nrxn1α homozygote KO mice and female Nrxn1α heterozygote mice (genotype effect: F(2,64)=16.51, p<0.0001; see Table S1).

**References**

1. Lad HV, Liu L, Paya-Cano JL, Parsons MJ, Kember R, et al. (2010) Behavioural battery testing: evaluation and behavioural outcomes in 8 inbred mouse strains. Physiol Behav 99: 301-316.

2. Prut L, Belzung C (2003) The open field as a paradigm to measure the effects of drugs on anxiety-like behaviors: a review. Eur J Pharmacol 463: 3-33.

3. Crawley J, Goodwin FK (1980) Preliminary report of a simple animal behavior model for the anxiolytic effects of benzodiazepines. Pharmacol Biochem Behav 13: 167-170.

4. Lister RG (1987) The use of a plus-maze to measure anxiety in the mouse. Psychopharmacology (Berl) 92: 180-185.

5. Hall CS (1934) Emotional behavior in the rat: Defecation and urination as measures of individual differences in emotionality. HJournal of comparative psychology 18: 19.

6. MIsslin RR, P. (1981) Effects of lateral amygdala lesions on the responses to novelty in mice. Behavioural processes 6: 8.

7. van Gaalen MM, Steckler T (2000) Behavioural analysis of four mouse strains in an anxiety test battery. Behav Brain Res 115: 95-106.

8. Morris R (1984) Developments of a water-maze procedure for studying spatial learning in the rat. J Neurosci Methods 11: 47-60.

9. Lipp HP, Wolfer DP (1998) Genetically modified mice and cognition. Curr Opin Neurobiol 8: 272-280.

10. D'Hooge R, De Deyn PP (2001) Applications of the Morris water maze in the study of learning and memory. Brain Res Brain Res Rev 36: 60-90.

11. van der Staay FJ (2000) Effects of the size of the morris water tank on spatial discrimination learning in the CFW1 mouse. Physiol Behav 68: 599-602.

12. Fernandes C, Hoyle E, Dempster E, Schalkwyk LC, Collier DA (2006) Performance deficit of alpha7 nicotinic receptor knockout mice in a delayed matching-to-place task suggests a mild impairment of working/episodic-like memory. Genes Brain Behav 5: 433-440.

13. Chen G, Chen KS, Knox J, Inglis J, Bernard A, et al. (2000) A learning deficit related to age and beta-amyloid plaques in a mouse model of Alzheimer's disease. Nature 408: 975-979.

14. Zeng H, Chattarji S, Barbarosie M, Rondi-Reig L, Philpot BD, et al. (2001) Forebrain-specific calcineurin knockout selectively impairs bidirectional synaptic plasticity and working/episodic-like memory. Cell 107: 617-629.

15. Yang M, Silverman JL, Crawley JN (2011) Automated three-chambered social approach task for mice. Curr Protoc Neurosci Chapter 8: Unit 8 26.

16. Winslow JT (2003) Mouse social recognition and preference. Curr Protoc Neurosci Chapter 8: Unit 8 16.

17. Etherton MR, Blaiss CA, Powell CM, Sudhof TC (2009) Mouse neurexin-1alpha deletion causes correlated electrophysiological and behavioral changes consistent with cognitive impairments. Proc Natl Acad Sci U S A 106: 17998-18003.

18. Lijam N, Paylor R, McDonald MP, Crawley JN, Deng CX, et al. (1997) Social interaction and sensorimotor gating abnormalities in mice lacking Dvl1. Cell 90: 895-905.

19. Peripato AC, Cheverud JM (2002) Genetic influences on maternal care. Am Nat 160 Suppl 6: S173-185.

20. Yang M, Crawley JN (2009) Simple behavioral assessment of mouse olfaction. Curr Protoc Neurosci Chapter 8: Unit 8 24.

21. Elvevag B, Goldberg TE (2000) Cognitive impairment in schizophrenia is the core of the disorder. Crit Rev Neurobiol 14: 1-21.

22. Wing L (1981) Language, social, and cognitive impairments in autism and severe mental retardation. J Autism Dev Disord 11: 31-44.

23. Association AP (2000) Diagnostic and statistical manual of mental disorders. Washington, DC.

**Table S1. Results from the novel object recognition, grooming and buried food tasks for the Nrxn1α WT, HET and KO mice.** Values are mean (± sem) for the time spent exploring the two objects for trials 1 and 2, and for both short-term memory (STM) and long-term memory (LTM) in the object recognition task, grooming and rearing behaviours in the grooming task, and latency to find the hidden food in the buried food task. Data is derived from 23 WT (12**♂**, 11**♀**) 29 HET (15**♂**, 14**♀**) 18 KO (9**♂**, 9**♀**) mice, and levels of significance indicated by ** and *** as p<0.01 and p<0001, respectively, compared to WT mice.

| **Task** | **Behaviour** | **WT** | | **HET** | | **KO** | |
| --- | --- | --- | --- | --- | --- | --- | --- |
|  |  | **Male** | **Female** | **Male** | **Female** | **Male** | **Female** |
| **Novel object task** | **Trial 1 STM – total distance travelled (cm)** | 2576.9 (±224.0) | 3256.8 (±141.8) | 3377.3 (±162.5) | 2939.8 (±122.9) | 2555.1 (±188.6) | 2162.2 (±160.8) |
|  | **Trial 1 LTM – total distance travelled (cm)** | 2086.1 (±96.9) | 2280.6 (±108.7) | 2343.4 (±103.4) | 2507.3 (±154.4) | 2387.2 (±208.3) | 1978.7 (±103.3) |
|  | **Trial 1 STM – time spent exploring objects** | 49.1 (±12.2) | 50.0 (±10.9) | 47.3 (±6.4) | 52.5 (±9.8) | 49.7(±8.1) | 34.3 (±9.2) |
|  | **Trial 1 LTM – time spent exploring objects** | 29.6 (±2.3) | 39.9 (±6.4) | 34.8 (±6.4) | 40.4 (±5.5) | 51.0 (±7.0) | 37.7 (±7.0) |
|  | **Trial 2 STM - Time spent exploring novel object (s)** | 42.1(±4.0) | 43.4(±5.5) | 49.4(±4.2) | 39.0(±3.6) | 51.1(±5.0) | 47.5(±4.2) |
|  | **Trial 2 STM - Time spent exploring familiar object (s)** | 45.4(±7.1) | 50.1(±4.4) | 37.3(±5.2) | 38.5(±4.0) | 41.2(±3.7) | 47.9(±6.3) |
|  | **Trial 2 LTM - Time spent exploring novel object (s)** | 33.2(±2.7) | 37.8(±4.5) | 35.2(±3.4) | 35.9(±4.0) | 37.6(±3.4) | 39.7(±3.0) |
|  | **Trial 2 LTM - Time spent exploring familiar object (s)** | 35.6(±3.6) | 41.6(±4.7) | 25.8(±2.5) | 34.7(±3.3) | 38.8(±5.2) | 43.4(±10.5) |
| **Grooming task** | **Time spent grooming (s)** | 40.1(±14.8) | 40.9(±8.2) | 35.2(±7.7) | 24.3(±6.9) | 25.0(±10.4) | 30.9(±12.2) |
|  | **No. of bouts of grooming** | 3.6(±1.0) | 5.0(±0.7) | 3.8(±0.8) | 2.9(±0.3) | 2.9(±0.7) | 5.6(±1.6) |
|  | **No. of rears** | 75.2(±4.1) | 62.4(±2.9) | 93.3(±5.5) | 89.4(±4.3) ** | 100.2(±5.7) *** | 89.9(±5.7) *** |
| **Buried food task** | **Latency to find the food (s)** | 19.2 (±4.8) | 46.0 (±14.7) | 15.5 (±1.6) | 46.0 (±11.8) | 22.6 (±6.3) | 42.7 (±6.9) |
